# Non-destructive detection of microplastics stress in rice seedling: an interpretable deep learning approach using excitation emission matrix fluorescence spectra of root exudates

**DOI:** 10.3389/fpls.2025.1653451

**Published:** 2025-12-02

**Authors:** Chaojie Wei, Hongxin Xie, Wei Wang, Yu-Feng Li, Xiaorong Wang, Ziwei Song, Fajun Chen

**Affiliations:** 1 Zhongyuan Petersburg Aviation College, Zhongyuan University of Technology, Zhengzhou, China; 2 CAS-HKU Joint Laboratory of Metallomics on Health and Environment, Beijing Metallomics Facility, National Consortium for Excellence in Metallomics, Institute of High Energy Physics, Chinese Academy of Sciences, Beijing, China; 3 CAS Key Laboratory for Biomedical Effects of Nanomaterials and Nanosafety, Beijing Metallomics Facility, National Consortium for Excellence in Metallomics, Institute of High Energy Physics, Chinese Academy of Sciences, Beijing, China; 4 College of Engineering, China Agricultural University, Beijing, China

**Keywords:** deep learning, excitation emission matrix fluorescence spectra, microplastics, rice seedling, root exudates

## Abstract

**Introduction:**

Microplastics (MPs), ubiquitous and insidious pollutants pervading agricultural systems, pose an escalating threat to global food security. This makes the development of nondestructive methods for the early detection of MPs stress in rice seedling an urgent scientific imperative.

**Method:**

Rice seedlings were cultivated under exposure to polyethylene terephthalate (PET), polystyrene (PS), and polyvinyl chloride (PVC) MPs at concentrations of 0 (control), 10, and 100 mg/L. Based on the stress-induced alterations in root exudates composition, a novel detection method for MPs stress in rice seedlings was developed using excitation-emission matrix fluorescence (EEMF) spectra combined with deep learning.

**Results:**

Analysis of the original EEMF spectra revealed discernible differences. Feature extraction was performed using both the peak method and the PARAFAC method. Spectral changes in seedlings exposed to the low MP concentration (10 mg/L) were relatively minor compared to the control group. In contrast, exposure to the high concentration (100 mg/L) induced significant alterations in humic acid-like and amino acid-like substances. Subsequently, enhanced Vision Transformer (VIT) models were developed utilizing three distinct data representations: full EEMF spectra, emission spectra at specific excitation wavelengths, and extracted characteristic fluorescence values. The optimal model achieved 100% classification accuracy. Furthermore, SHapley Additive exPlanations (SHAP) analysis was employed to evaluate feature importance, identifying both humic acid-like and marine humic acid-like components as major contributors to the model’s predictions.

**Conclusion:**

In summary, this study establishes a novel, non-destructive, and interpretable framework for the early detection of MPs stress in rice seedlings based on EEMF spectra of root exudates combined with deep learning.

## 1 Introduction

In recent years, microplastics (MPs) pollution has become a global problem, and its potential health risks garnering unprecedented attention (Zhang et al., 2022). Among affected ecosystems, agricultural systems have become significant accumulation sites for MPs (Yadav et al., 2022). Rice, as a critical global food crop, directly influences food security and human well-being through its plant health (Edwards et al., 2024). thus, the safety of its growth environment is paramount. The threat of MPs to crops like rice constitutes an understudied endogenous stress (Mamathaxim et al., 2023). Researches show these particles (< 5 mm) accumulate in soil/water, impairing roots development, inhibiting water/nutrient uptake, inducing oxidative stress, damaging cell structures, and ultimately reducing biomass, yield, and quality (Yu et al., 2021). Critically, rice can absorb MPs and associated toxins (e.g., heavy metals, persistent organic pollutants), enabling transfer into grains via the food chain (Cao et al., 2021). Human consumption of contaminated rice poses severe long-term risks, including bioaccumulation in organs and exposure to carcinogenic/mutagenic additives that disrupt endocrine systems (Tang et al., 2024). Because the seedling stage represents rice’s most stress-sensitive life phase, early detection of MPs stress at this juncture holds critical scientific and practical significance.

At present, microscopes, mass spectrometry, spectroscopy and other emerging technologies offer high sensitivity for MPs and enable *in-situ* analysis (Ye et al., 2022). However, their destructive sampling, complex pretreatment and technical expertise requirements hider non-destructive plant monitoring. Root exudates specifically refer to various organic substances released by plants into the rhizosphere environment through active metabolism or passive exudation (Vives-Peris et al., 2020). Studies have shown that when plants are exposed to abiotic stresses such as heavy metal pollution and MPs invasion, root exudates will show specific changes (Chai and Schachtman, 2022). These dynamic responses range from pollutant detoxification through chelation to antimicrobial compound release, reflecting plants’ rhizosphere-modulating survival strategies (Huang et al., 2014). Notably, there have been studies exploring the effects of polystyrene (PS), polyethylene (PE), and polypropylene (PP) MPs on the root exudates of tomatoes under hydroponic conditions (Shi et al., 2023). It has been found that MPs promoted the secretion of organic acids, malic acid, and myristic acid, etc. PS MPs stress lettuce leads to an increase in the biosynthesis of ascorbic acid, terpenoids and flavonoids in root exudates (Wang et al., 2023). MPs stress interferes with the normal metabolic process of root secretions in tomato (Shi et al., 2023), resulting in abnormalities of substances such as amino acids, organic acids and phenolic compounds to cope with adverse stress. This would provide the possibility for detecting MPs stress with root exudates as characteristic markers.

Excitation emission matrix fluorescence (EEMF) spectra is a novel fluorescence analysis technology capturing fluorescence intensity across simultaneous excitation/emission wavelength scans, exceling at characterizing multi-component mixtures (Wei et al., 2025). It detects stress-induced fluorescent-substance changes in root exudates, revealing physiological states of MPs-stressed rice seedlings (Fan et al., 2025) (Zhu et al., 2019). studied the phenomenon of metal complexation between root exudates of avicennia marina by EEMF spectra and found that the fluorescence intensity of fulvic acid increased with the increase of external salt content (Liu et al., 2018). analyzed the soluble organic components extracted from large plants using EEMF spectra technology and found that they mainly contained fulvic acid, humus-like and protein-like fluorescent substances. Therefore, EEMF-based monitoring of root exudate fluorescence offers significant promise for MPs stress detection.

To achieve non-destructive, accurate, and interpretable detection of MPs stress, this study employed machine learning and deep learning techniques to extract key features from EEMF spectra of rice seedling root exudates, construct detection models, and analyze feature importance. The peak method was first employed to rapidly identify characteristic response regions in MPs-stressed root exudates for preliminary spectral feature recognition (Komatsu et al., 2025). Subsequently, Parallel Factor Analysis (PARAFAC) decomposition was applied to resolve key fluorescent components within complex three-dimensional EEMF spectra, effectively isolating target signals (Murphy et al., 2013). To full leverage global spectral information and establish a high-precision detection model, a deep learning model is implemented for feature extraction and classification. Finally, the SHAP interpretability framework quantified features importance to clarify the decision-making pathways and identify the most indicative stress biomarkers (Choi et al., 2025).

This study aims to establish a non-destructive detection method for MPs stress in rice seedlings using EEMF spectra of root exudates. The specific objectives are to characterize MPs-induced variations in EEMF spectral profiles; extract characteristic fluorescent substances through feature extraction methods; develop a deep learning-based detection model for MPs-stressed seedlings, and quantify feature importance via explainable artificial intelligence analysis. To our knowledge, this represents the first investigation detecting rice seedlings under MPs stress through EEMF spectra of root exudates. The proposed methodology may extend to detecting other emerging environmental pollutants.

## 2 Materials and methods

### 2.1 Sample preparation

Microplastics of polyethylene terephthalate (PET), polystyrene (PS), and polyvinyl chloride (PVC), purchased from HengfaSuhua (Guangdong, China), were characterized using a dynamic light scattering (DLS) particle size analyzer (Zetasizer Nano ZS90, UK), revealing a particle size distribution concentrated within 3-6 μm. Rice (Liangyou Y900) was cultivated according to the method of (Xie et al., 2024), whereby fifty seeds were placed in each glass petri dish and subsequently treated with suspensions of PET, PS, or PVC MPs at concentrations of 10 mg/L or 100 mg/L, while rice cultivated with sterile water served as the control group. Six replicates were prepared for each treatment. Under 7 days dark conditions, 5 mL of the respective MP suspension or sterile water was added to each dish daily for rice germination. Then, the seedlings were transferred to an environment with a 14 h light/10 h dark photoperiod for further cultivation over 5 weeks. At the end of 5 week, seedlings were removed from the hydroponic solution, and their roots were thoroughly rinsed with deionized water. Subsequently, the rinsed rice seedlings were transferred to conical flasks containing 50 mL of sterile water and left to stand in darkness for 4-6 hours to collect of root exudates.

### 2.2 Collection of EEMF spectra

The EEMF spectra of root exudates were collected using a Shanghai Lingguang F98 fluorescence spectrometer equipped with a 150 W xenon lamp and a standard right-angle optical assembly. Instrument parameters were configured as follows: excitation wavelengths ranged 220–550 nm at 5 nm intervals, emission wavelengths ranged 240–750 nm at 1 nm intervals, the scan speed was set to 30,000 nm/min, and the pulse width modulation (PMT) voltage was maintained at 850 V. 3-4 mL root exudates was transferred into quartz colorimetric dish using a disposable syringe to obtain EEMF spectra. The scanning time for a single sample is approximately 4 minutes. To reduce noise interference, three repeat measurements were performed per sample. EEMF spectra were collected for 48 root exudates per experimental group, yielding a total of 336 spectra.

**Figure 1:**
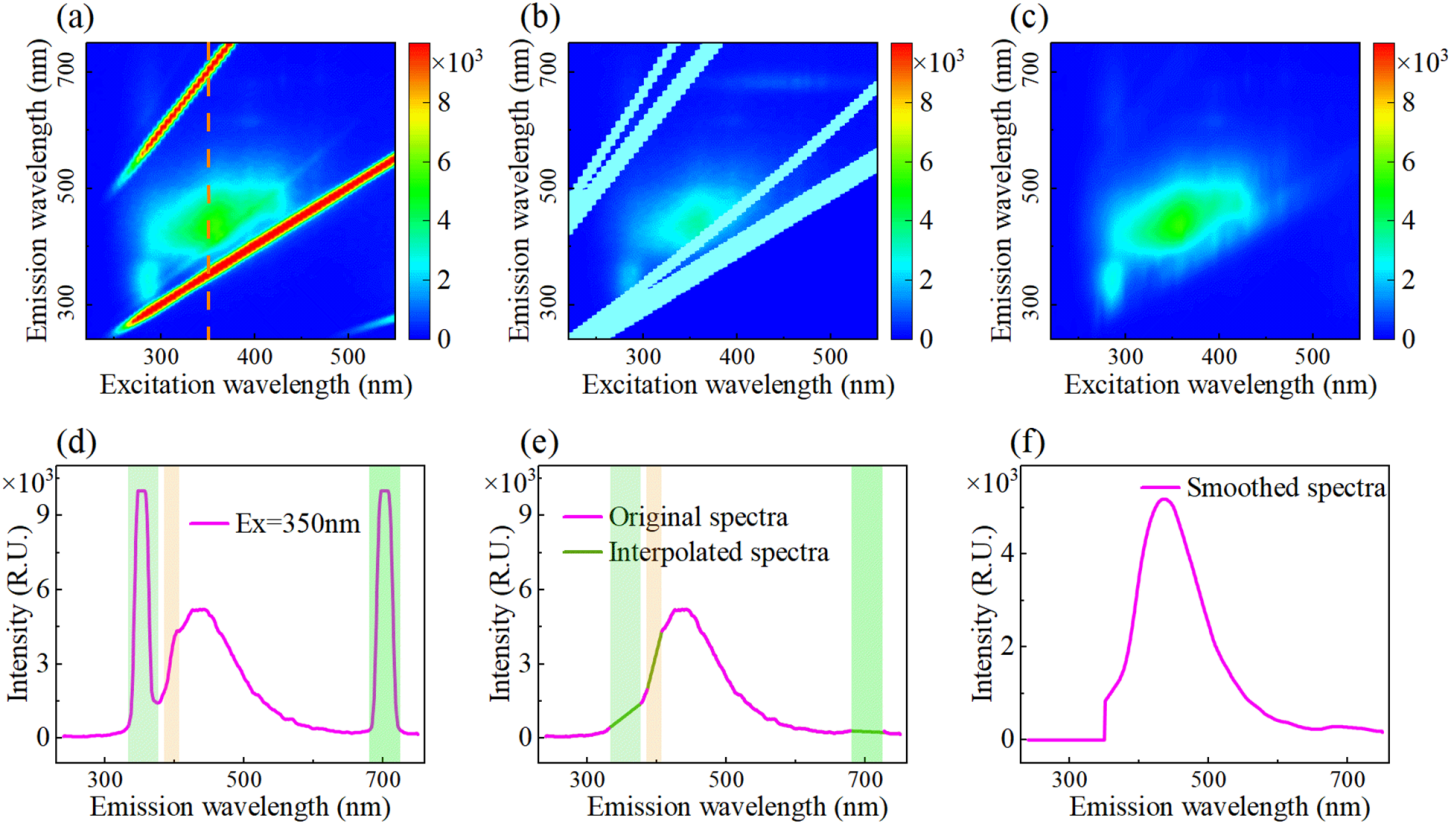
Pretreatment for removing fluorescence scattering peaks. **(a)** Original EEMF spectra, **(b)** Remove scattering, **(c)** Fitted EEMF spectra, **(d)** Original emission spectra, **(e)** Scattering removal and interpolated spectra, and **(f)** Smoothed spectra.

Rayleigh and Raman scattering (Engelen et al., 2007) existed in EEMF spectra due to molecular polarization induced by incident light, and scatter removal was performed (**Figure 1**). As shown in **Figure 1a**, Raman scattering occurs at Stokes/anti-Stokes lines, which caused by inelastic collisions between photons and molecular vibrations/rotations. Rayleigh scattering exhibiting the same wavelength as the excitation light originate from elastic collisions, which is intense but lacks chemical information. Second-order scattering signals of both are weak. Fluorescence intensity values within the scatter regions were masked as Not a Number (NaN), as depicted in **Figure 1b**. Subsequently, linear interpolation and moving average smoothing were applied to these regions. Furthermore, since emitted photons possess lower energy than excitation photons, the region where emission wavelength is less than excitation wavelength was set to zero (**Figure 1c**). Meanwhile, the emission spectrum at an excitation wavelength of 350 nm is presented to demonstrate the one-dimensional spectral processing. The original emission spectrum (**Figure 1d**) exhibited first-order Rayleigh scattering near 350 nm, first-order Raman scattering near 400 nm, and second-order Rayleigh scattering near 700 nm. Results after scatter removal, linear interpolation, and mean filtering are shown in **Figure 1e**). Finally, regions where emission wavelengths below 350 nm were set to zero, yielding the smoothed emission spectrum (**Figure 1f**).

### 2.3 Feature extraction

#### 2.3.1 Peak method

Peak method extracts the fluorescence intensity of specific peak locations/regions and characterizes fluorescence components of EEMF spectra (Komatsu et al., 2025). In this study, the EEMF spectra of rice root exudates revealed multiple characteristic peaks, which were associated with complex organic compounds present, including amino acids, phenolic compounds, and humic acids. To analyze the fluorescence characteristics of the root exudates, seven primary fluorescent substances were selected for investigation. The locations of the respective fluorescence peaks and detailed information on their corresponding substances are presented in **Table 1**. The peak method effectively reveals variations in the content of different fluorescent components, making it an efficient approach for the rapid fingerprinting of root exudates.

**Table 1:** Characteristic fluorescence peaks and their assignments.

Peak	Excitation wavelength (nm)	Emission wavelength (nm)	Fluorescent substance
A	260	400-460	UV humic acid
M	290-310	370-410	Marine humic acid
C	320-360	420-460	Visible humic acid
D	390	509	Fulvic acid
B	275	305	Tyrosine-like compound
T	275	340	Tryptophan-like compound
N	280	370	Phytoplankton-related compound

#### 2.3.2 PARAFAC

Parallel Factor Analysis (PARAFAC) is applied to EEMF spectra to provide a robust analytical methodology for resolving complex fluorescence signatures within root exudates (Yu et al., 2019). The entire EEMF spectral dataset (sample × excitation wavelength × emission wavelength) is decomposed by PARAFAC into chemically interpretable components. This multi-channel technology addresses the spectral overlap of co-fluorescent moieties by extracting the pure excitation and emission loadings for each component, along with their relative concentrations in the samples. It is crucial that PARAFAC utilizes the intrinsic trilinear of EEMF data to achieve this separation without the need for prior assumptions about spectral distribution. The number of components is determined through split-half analysis and core consistency diagnostic. Therefore, complex spectral data are transformed by PARAFAC into quantitative fingerprints of root exudate composition, providing a powerful tool for investigating rhizospheric stress mechanisms in rice seedlings under MPs stress.

### 2.4 Models development, evaluation, and explanation

**Figure 2:**
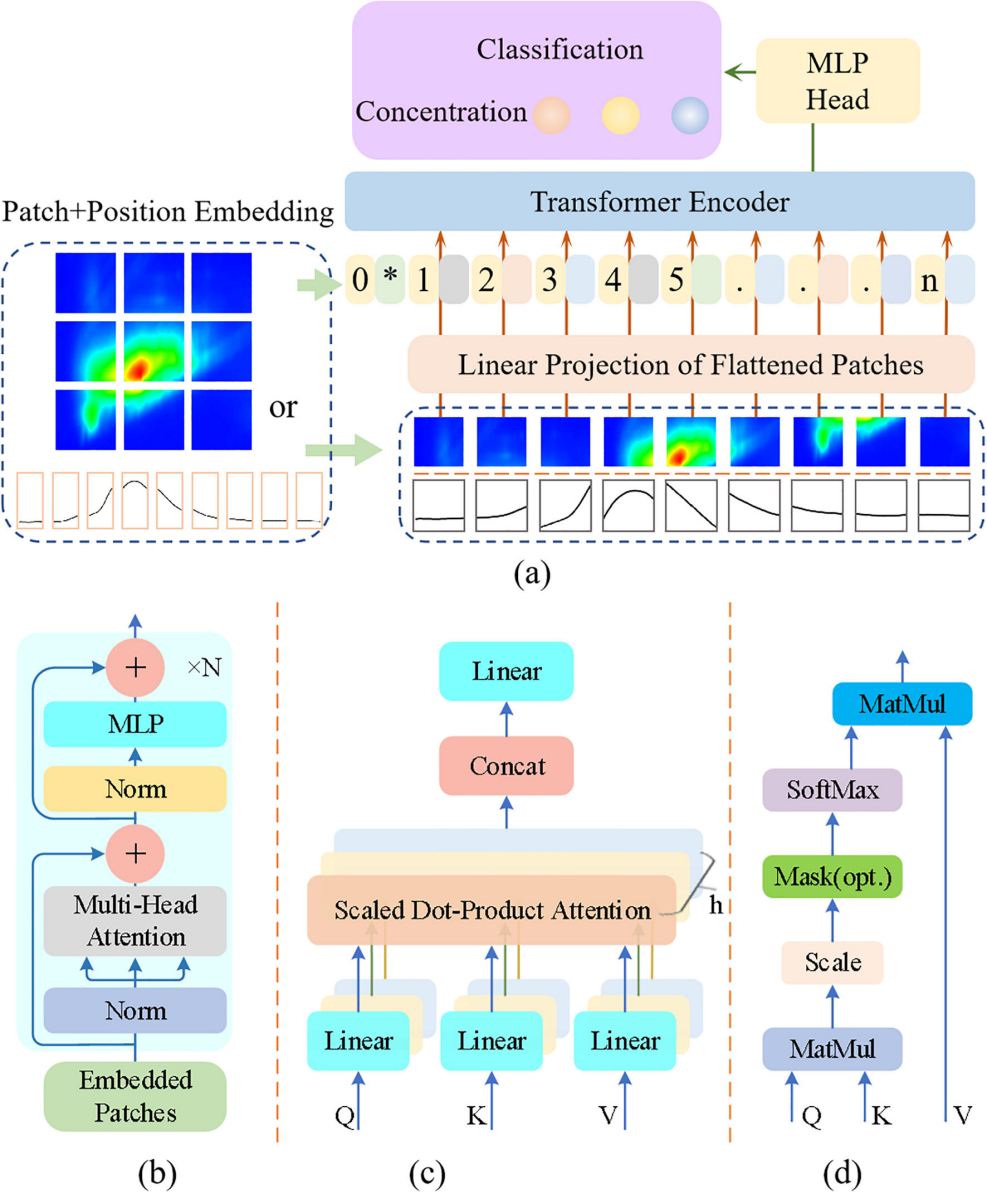
The framework of improved VIT model. **(a)** Proposed VIT model, **(b)** Transformer Encoder, **(c)** Multi-Head Attention, and **(d)** Scaled Dot-Product Attention.

The EEMF spectra consists of a two-dimensional matrix with excitation wavelengths ranging from 220-550 nm and emission wavelengths ranging from 240-750 nm, while the emission spectra at specific excitation wavelengths are one-dimensional spectral lines. In order to simultaneously process one-dimensional and two-dimensional data, an improved Vision Transformer (VIT) model was proposed for detecting MPs stress concentration (**Figure 2a**). The data is partitioned into fixed-size patches by the VIT model and global information is captured using self-attention mechanism. The data with both position information and slice vectors is input into the Transformer encoder (**Figure 2b**), which is mainly consists of two parts: Normalization (Norm) + Multi Head Attention, and Normalization + Multi-Layer Perceptron (MLP). Layer normalization is adopted by the improved model instead of batch normalization commonly used in convolutional neural networks, which can reduce fluctuations caused by differences in value ranges. The query (Q), key (K), and value (V) obtained through linear transformation of the input vector enter the Multi Head Attention (**Figure 2c**), which divides the model into multiple subspaces and simultaneously focuses on information at different levels. The Q, K, and V vectors are projected into the scaled dot-product attention module (**Figure 2d**) through linear full connection, which is the core component of the Transformer model and is essential for enabling parallel data processing. By calculating the dot product of Q and K, dividing by $\sqrt{d_{k}}$ to achieve scaling and ensure data stability, the Softmax function is used to convert the scaled data into a probability distribution to determine importance, and finally weighted and summed to obtain the output, as shown in Equation 1.

(1) $$Attention(Q,K,V)=softmax(\frac{QK^{T}}{\sqrt{d_{k}}})V$$

Where, Q, K, and V represent the query matrix, key matrix, and value matrix, respectively, and $d_{k}$ is the dimension of the key vector. The scaling factor $\frac{1}{\sqrt{d_{k}}}$ can avoid gradient vanishing and exploding problems, ensuring the stability of training.

The data processed by the Transformer encoder enters the Multi-Layer Perceptron head (MLP head), which is responsible for converting the encoder output into classification tasks. This module consists of a linear layer and an activation layer. The input features are dimensionally mapped to a higher space through the linear layer, and the activation layer with nonlinear functions acquires more complex expression capabilities. Dropout technique is used to prevent overfitting, and then mapped to the types of microplastic stress concentrations through linear full connections.

To evaluate the feasibility of the proposed VIT model, simple machine learning models (K-Nearest Neighbor (KNN)), and integrated machine learning models (Random Forest (RF)) were compared against it. Accuracy, specificity, and sensitivity were adopted as evaluation criteria (Zhu et al., 2022). SHAP, a game theory-based model interpretation framework, provides global and local interpretations by quantifying feature contributions to prediction outcomes (Choi et al., 2025). In this study, SHAP was employed to analyze the contribution of individual wavelengths to the model.

## 3 Results and discussion

### 3.1 EEMF spectral analysis

**Figure 3:**
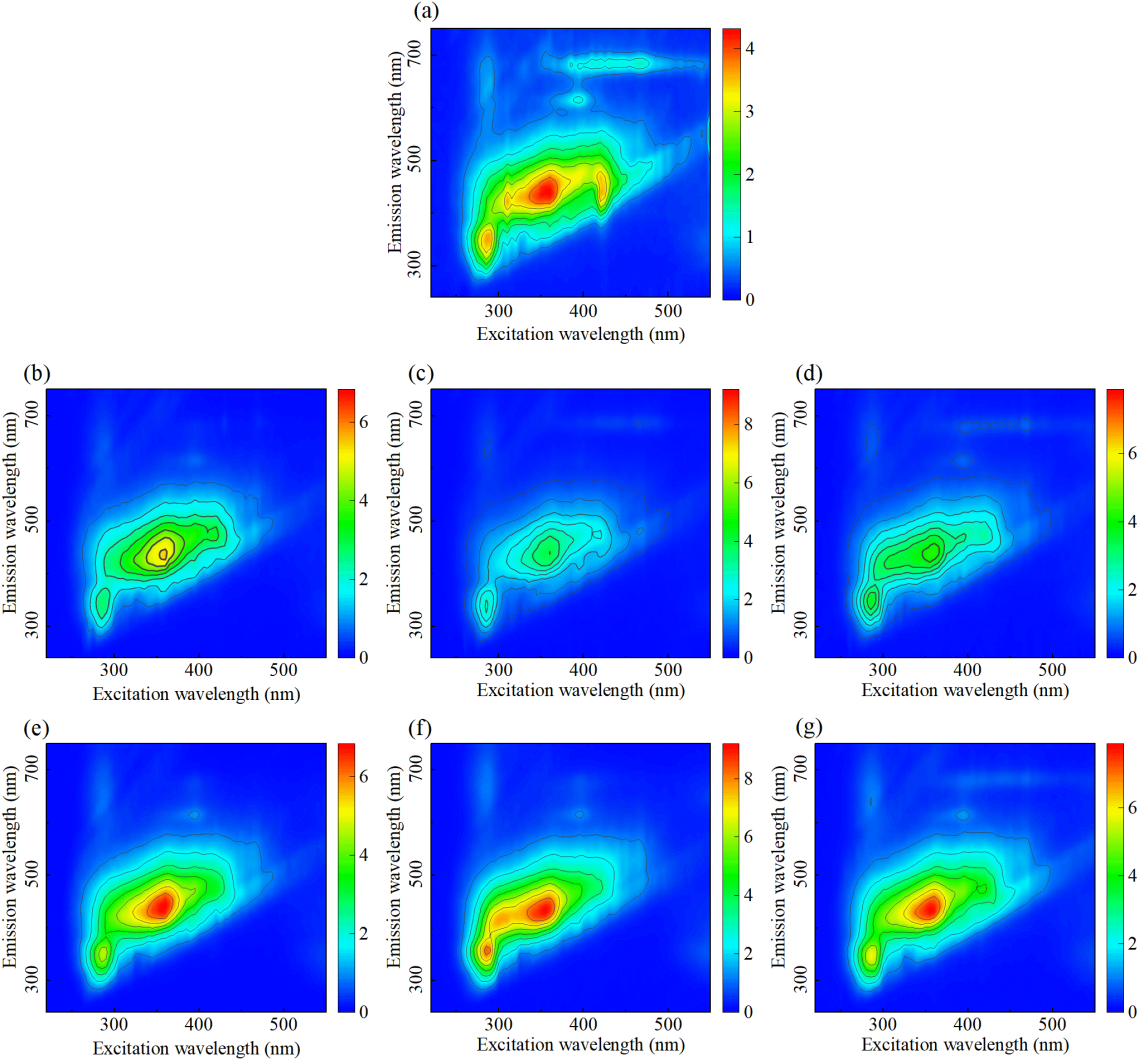
EEMF spectra of root exudates treated with different concentrations and types of MPs. **(a)** Control, **(b)** 10 mg/L PET, **(c)** 10 mg/L PS, **(d)** 10 mg/L PVC, **(e)** 100 mg/L PET, **(f)** 100 mg/L PS, and **(g)** 100 mg/L PVC.

**Figure 3** shows the EEMF spectra of root exudates of rice seedling under stress from different MPs concentrations and types. Three fluorescence peaks are identified within the raw spectra, with excitation/emission wavelengths centered at 285/345 nm, 355/438 nm, and 423/450 nm, corresponding to the tryptophan, visible humic acid, and pigment, respectively. The changes in amino acids and humic acid related root exudates provide a basis for detecting MPs stress. When the MPs concentration increased from 10 mg/L to 100 mg/L, the fluorescence intensity was enhanced, indicating that the stress response of rice seedlings was strengthened with the increase of MPs stress concentration. At a concentration of 10 mg/L, the EEMF spectra exhibited minimal changes across all three MP types, indicating negligible alterations in rice seedling root exudates under low-concentration stress. However, at 100 mg/L, the spectral shape remains consistent, but the fluorescence intensity is relatively higher. PS causes the highest fluorescence intensity, followed by PVC, with PET lowest. This might be related to the differences in the content of root exudates caused by different types of MPs. The characteristics such as the position, intensity, shape and width of the peaks in EEMF spectra are closely related to the chemical structure and properties of fluorescent substances (Park and Snyder, 2018). Therefore, it is necessary to carry out feature extraction and analysis.

### 3.2 Fluorescence feature analysis

#### 3.2.1 Feature extraction of peak method

**Figure 4:**
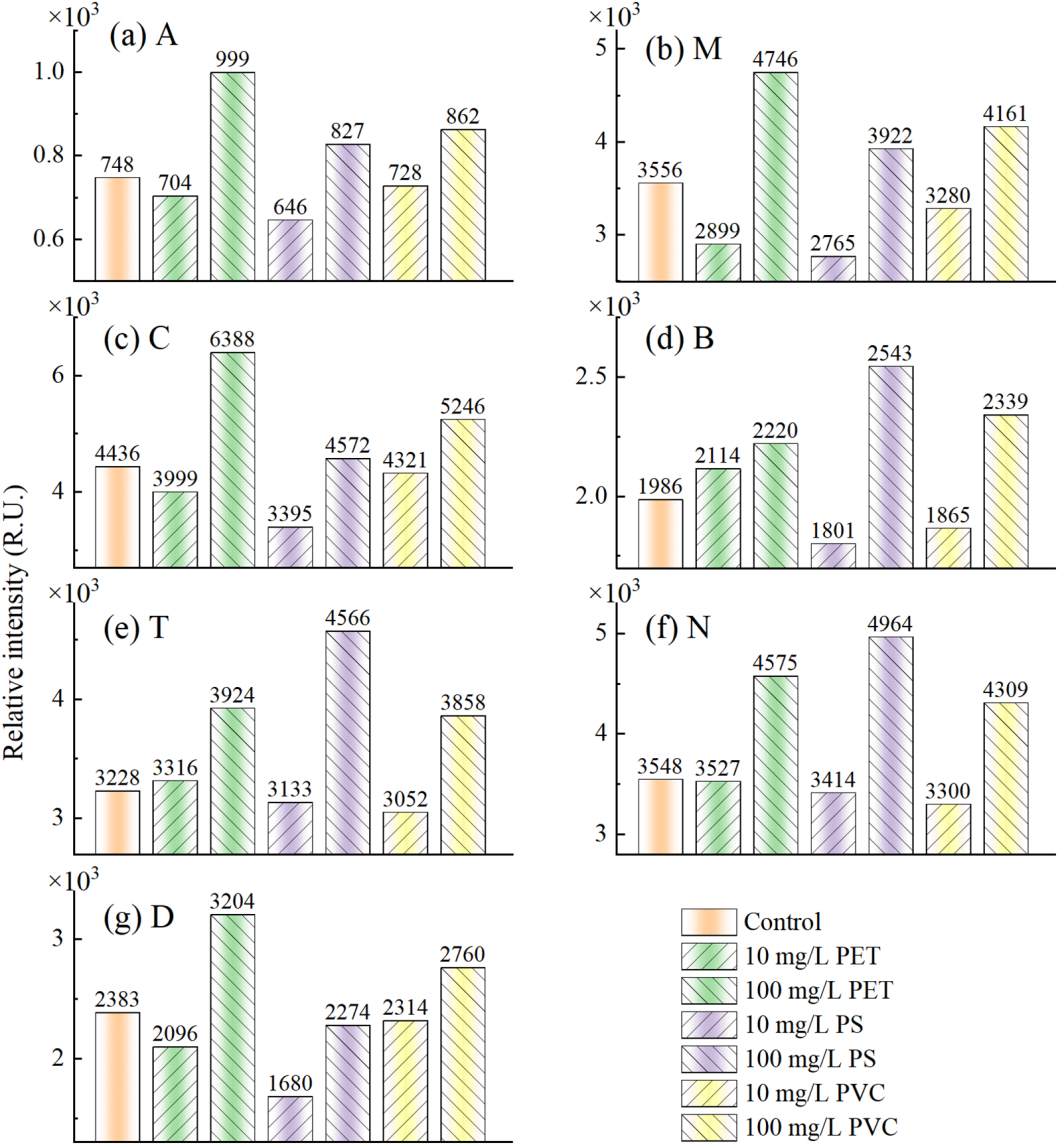
Feature extraction of peak method. (a) A, (b) M, (c) C, (d) B, (e) T, (f) N, and (g) D.

The intensity of fluorescence peaks was calculated using peak method, including UV humic acid (A, Ex/Em=260/400-460 nm), marine humic acid (M, Ex/Em=290-310/370-410 nm), visible humic acid (C, Ex/Em=320-360/420-460 nm), tyrosine like (B, Ex/Em=275/305 nm), tryptophan like (T, Ex/Em=275/340 nm), phytoplankton related (N, Ex/Em=280/370 nm), and fulvic acid (D, Ex/Em=390/509 nm). **Figure 4** shows the mean intensities of each fluorescence peak in the EEMF spectra of the seven sample groups. Among them, orange represents the control group, while green, purple, and yellow represent PET, PS, and PVC stress, respectively. Left and right sloping stripes represent the stress concentrations of 10 mg/L and 100 mg/L, respectively. Under low concentration (10 mg/L) stress, the fluorescence peak intensities of A, M, C, N, and D were all lower than those of the control group. The fluorescence peak intensities of B and T were higher than those of the control group under PET stress, while they were lower than those under PS and PVC stress. This indicates that low concentrations of PS and PVC have a weak inhibitory effect on the release of amino acid substances and may even promote their release. Under high concentration stress (100 mg/L), all three MP types exhibited elevated fluorescence peak intensities at regions A, M, C, B, T, and N compared to controls, indicating concurrent inhibition of root exudate production and enhanced spectral response. The fluorescence peak intensity of D increased under PET and PVC stress, while it decreased under PS stress. This phenomenon may be related to the benzene ring structure of PS promoting the antioxidant capacity of rice.

MPs stress changed the microbial community in the rhizosphere environment of rice seedlings, affecting the content of humic acid substances (A, M, C) in root exudates (Kang et al., 2023). Tryptophan (T) and tyrosine (B) are respectively involved in the synthesis of bioactive substances such as bioactive amines, plant hormones, proteins and dopamine. MPs stress affects the nutritional status of rice, leading to abnormal synthesis and metabolism of amino acid substances. Phytoplankton related substances (N) may be associated with phenolic compounds, pigments, and organic acids with antioxidant effects (Lebel et al., 2025). Rice promotes the exudates of phenolic substances and flavonoids, enhancing the ability to resist oxidative stress and environmental pressure. Fulvic acid (D), as an important organic component in humus, has the functions of regulating pH, promoting plant growth, eliminating free radicals and delaying cell aging (Cai et al., 2024). MPs stress inhibited the metabolic activities of rice cells, resulting in a reduction in the synthesis and accumulation of fulvic acid (D) in the root environment. In addition, MPs stress also induces rice seedling to secrete more organic acids, phenolic compounds and other secondary metabolites to cope with environmental stress.

Overall, low-concentration MPs stress promotes the production of rice roots exudates, resulting in decrease in the intensity of fluorescence peaks. However, high-concentration stress inhibits the stress response of rice and the production of root exudates, thereby enhancing the intensity of the fluorescence peak. This result indicates that rice roots exudates have a significant impact on the fluorescence characteristics of MPs stress concentrations, laying the foundation for rapid detection.

#### 3.2.2 Feature extraction of PARAFAC

Due to the complex composition of root exudates, the highly overlapping EEMF spectra increase the difficulty of direct analysis and quantitative analysis. PARAFAC, a multidimensional decomposition method, resolves complex fluorescence signals into independent factors, enabling direct identification and quantification of specific compounds in samples. The residual variation trends under 3-6 factors were compared, and the error was minimized when the factor was 5. In addition, Split half analysis shows that the similarity of PARAFAC with 5 factors is greater than 95%, therefore the 5 factors are the optimal results.

**Figure 5:**
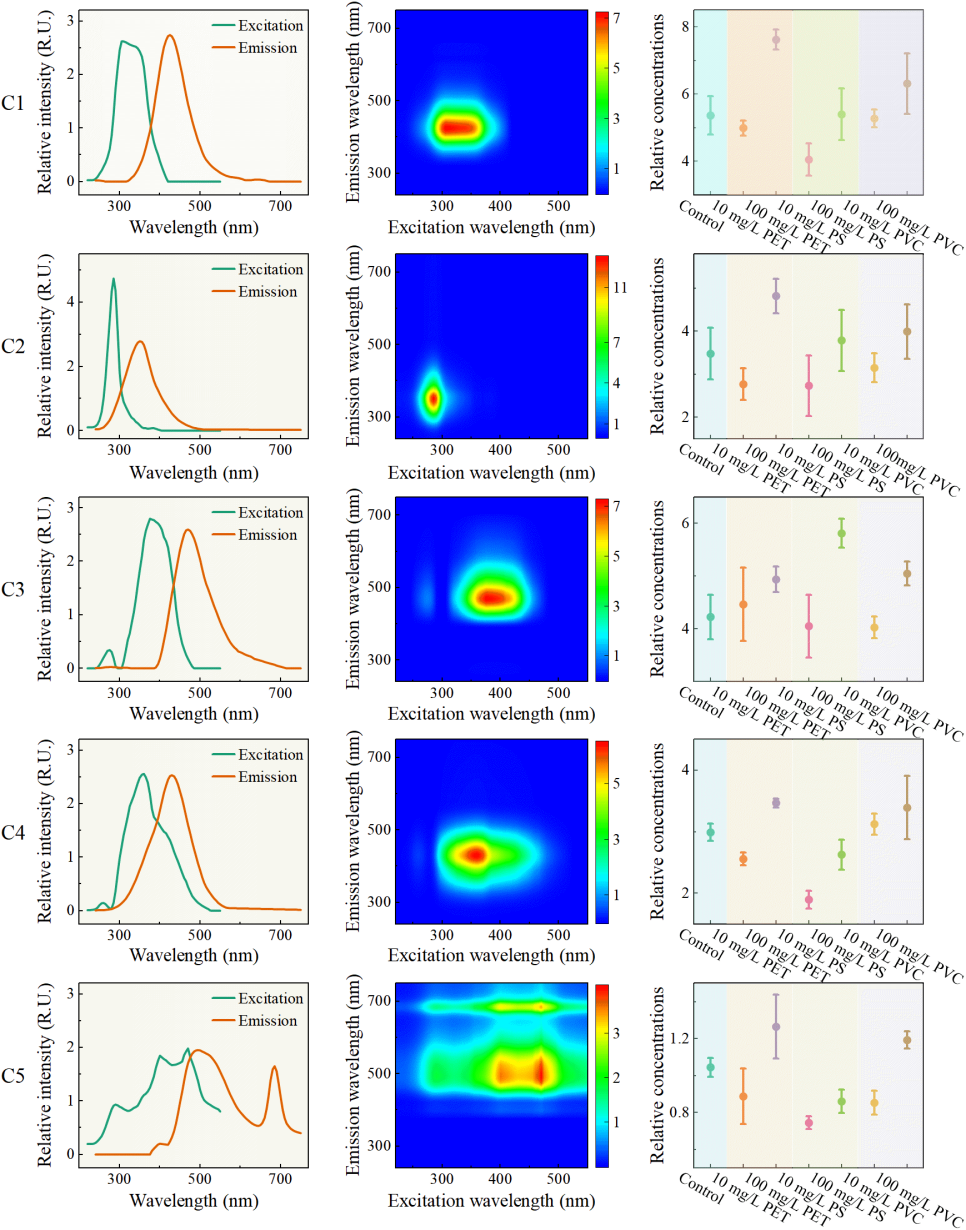
Feature extraction of PARAFAC method.

Five fluorescent components (labeled as C1-C5) obtained by PARAFAC are shown in **Figure 5**. The excitation/emission peak of C1 is located at 305 nm/425 nm, which may be related to tryptophan (Cory and McKnight, 2005). When plants are subjected to environmental stress, it usually promotes the exudates of tryptophan by the plant roots to increase the fluorescence intensity. Tryptophan and its derivatives play significant roles in plant antioxidation and signal transduction, which may indicate that rice roots enhance their stress resistance by regulating metabolism. The excitation/emission peak of C2 is at 285 nm/355 nm, which is related to protein-like substances such as tyrosine or phenylalanine (D’Andrilli et al., 2019). Rice roots mitigate microplastic-induced damage by restructuring the rhizosphere microbial community through enhanced protein secretion, thereby stabilizing the root environment. The excitation/emission peak of C3 is at 375 nm/475 nm, which may be related to humic acid (Lin and Guo, 2020). Humic acid enhances the adsorption capacity of MPs and reduces the direct contact of MPs with root. The excitation/emission peak of C4 is at 360 nm/430 nm, which may be related to fulvic acid (Cai et al., 2024). Fulvic acid with high biological activity, promotes the absorption and utilization of nutrients by roots, and alleviates the inhibitory effect of MPs stress on growth. In addition, fulvic acid also enhance the metabolic activity of rhizosphere microorganisms to improve the health status of the rhizosphere environment. The excitation wavelengths of C5 are 290 nm, 400 nm and 470 nm respectively, and the emission wavelengths are 495 nm and 685 nm. This multi excitation/emission wavelength characteristic indicates that C5 is a complex fluorescent component, which may contain the superposition of flavonoids, humus-like substances, fulvic acid-like substances, and residual fluorescent substances.

The variation of relative concentrations (fluorescence intensity) of EEMF spectra is shown in the third column of **Figure 5**. Relative concentrations of each component increased with the increase of MPs concentration from 10 mg/L to 100 mg/L, which reflects that MPs promote the accumulation of various fluorescent substances in root exudates. In most cases, the relative concentrations of low concentration stress were lower than that of the control group, while the relative concentrations of high concentration were higher than that of the control group, except in some special cases. This phenomenon is consistent with the result of the peak method analysis, which provides a basis for identifying MPs stress based on the changes in the fluorescent components of the root secretions of rice seedlings.

### 3.3 Microplastics detection models

In order to achieve rapid detection of MPs stress, detection models were developed respectively using EEMF spectra, emission spectra of characteristic excitation wavelengths, and characteristic variables based on peak method and PARAFAC.

#### 3.3.1 Detection models of EEMF spectra

Three representative models ranging from simple to complex, KNN, RF and VIT, were adopted to predict the MPs stress concentration, and the results are shown in **Table 2**. The EEMF spectra is reshaped into one-dimensional vectors and input into the KNN and RF models for model establishment, and the two-dimensional matrix of EEMF spectra is used for VIT model development. As a simple model, the accuracy rate of KNN in concentration detection is 93.85-98.32%. RF, as an ensemble learning model, demonstrates strong robustness, with an accuracy rate ranging from 97.77 to 99.44%. The most complex VIT model performs the best, and the accuracy rate of the validation set generally exceeds 98.88%, fully demonstrating its advantages. Overall, models ranging from simple to complex all demonstrated high classification performance, and the model complexity showed a positive correlation trend with the accuracy rate. These results confirm that EEMF spectra of root exudates effectively distinguish MP stress concentrations and demonstrate the discriminatory capability of the proposed VIT model.

**Table 2:** Detection accuracy of different models based on EEMF spectra.

MPs types	Model	Calibration set	Cross validation	Validation set
PET	KNN	99.52	97.77	98.32
	RF	100	98.88	99.44
	VIT	100	100	100
PS	KNN	99.28	98.11	98.32
	RF	99.76	97.92	98.88
	VIT	100	98.78	99.44
PVC	KNN	96.39	94.25	93.85
	RF	98.80	98.00	97.77
	VIT	99.28	97.67	98.88

#### 3.3.2 Detection model of characteristic excitation wavelength

**Figure 6:**
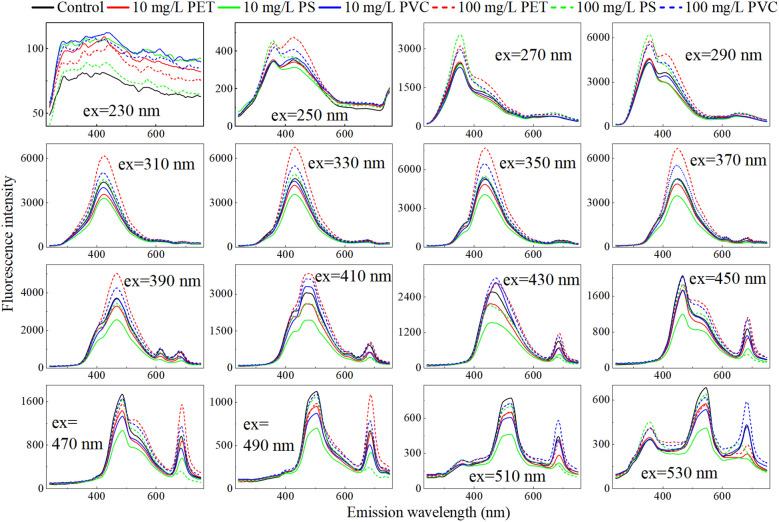
Emission spectra at different excitation wavelengths.

Due to the huge amount of EEMF spectra data, a single sample contains 34,237 parameters (excitation wavelength 67× emission wavelength 511), which poses a huge challenge for rapid detection. The excitation wavelength can excite the fluorescence in the sample and directly affect the generation and detection of fluorescence. Therefore, determining the excitation wavelength characterizing root exudates is a key step in actual detection. **Figure 6** shows the emission spectra at the excitation wavelength with an excitation interval of 20nm. With the increase of the excitation wavelength, the maximum intensity of the fluorescence peak gradually increases. When the excitation wavelength is 350 nm, the fluorescence peak reaches its strongest and then gradually weakens. Further analysis revealed elevated fluorescence intensity in the high-concentration MP group relative to controls across 250-410 nm excitation wavelengths, while the low-concentration group showed depressed intensity. Between 410 nm and 450nm, the fluorescence intensities of the high-concentration and low-concentration groups crossed. When the excitation wavelength is greater than 450 nm, the control group is at the top, the low-concentration group is in the middle, and the high-concentration group is at the bottom. This regular change indicates that the selection of excitation wavelengths has an important influence on distinguishing different MPs stress concentrations.

**Figure 7:**
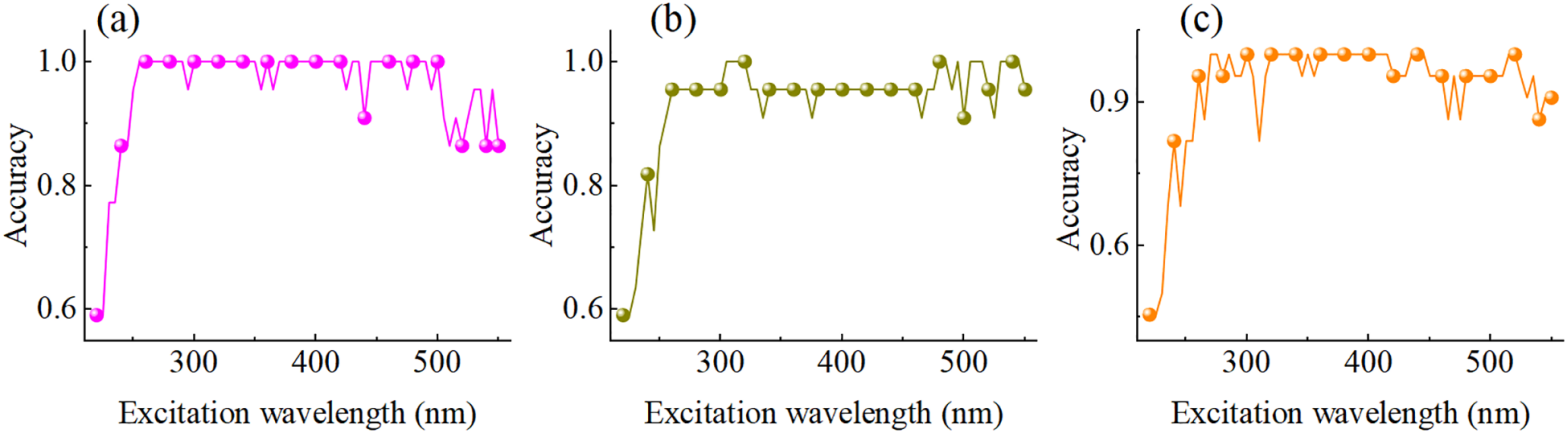
Detection accuracy of models under different excitation wavelengths. **(a)** PET, **(b)** PS, and **(c)** PVC.

Based on the emission spectra at different excitation wavelengths ranging from 220 nm to 550 nm, a VIT model for classifying the MPs stress concentration was established and the accuracy variation trend of the validation set was analysed. The results are shown in **Figure 7**. Overall, the accuracy rates of the three MPs prediction models show a consistent pattern with the variation of excitation wavelengths. From 220 nm to 300 nm, the accuracy gradually increases with the increase of the excitation wavelength. It achieves a relatively high predictive ability within the range of 300 nm to 450 nm, approaching or reaching 100% multiple times. Especially around 350 nm, the accuracy rates of the three MPs detection were significantly close to the peak, indicating that 350 nm might be the key excitation wavelength for differentiating MPs stress.

#### 3.3.3 Detection models of fluorescence feature

Peak method and PARAFAC extracted 7 and 5 features, respectively, as inputs for the model to distinguish the MPs stress concentration. The results are shown in **Table 3**. Both methods demonstrated high classification performance in the detection of three MPs types, but there were certain differences in accuracy and stability. The peak method demonstrated perfect classification performance in detection of stress concentration. The specificity, sensitivity and accuracy were all 100%, indicating that it could completely distinguish MPs stress of 0, 10 and 100 mg/L. The specificity of the PARAFAC method in PET detection was 97.92-100%, the sensitivity was 95.83-100%, the accuracy rate was 98.61%, and only one sample was misjudged. The specificity of PS MP detection was 87.5-100%, the sensitivity was 87.5-100%, and the accuracy rate was 91.67%. There are cases where samples of 0 and 10 mg/L are mistakenly judged as 100 mg/L. In PVC MP detection, there were two samples of 0 mg/L that were misjudged as 10 mg/L, but still maintained relatively high classification performance. The peak method demonstrated excellent performance in concentration detection, with specificity, sensitivity and accuracy generally reaching 100%. Although the overall performance of the PARAFAC method was slightly lower than that of the peak method, its accuracy still remained between 91.67% and 98.61%.

**Table 3:** Stressconcentration prediction based on peak method and PARAFAC.

MPs types	Concentration	Peak			PARAFAC		
		0 mg/L	10 mg/L	100 mg/L	0 mg/L	10 mg/L	100 mg/L
PET	0 mg/L	24	0	0	23	1	0
	10 mg/L	0	24	0	0	24	0
	100 mg/L	0	0	24	0	0	24
	Specificity (%)	100	100	100	100	97.92	100
	Sensitivity (%)	100	100	100	95.83	100	100
	Accuracy (%)	100			98.61		
PS	0 mg/L	24	0	0	21	0	3
	10 mg/L	0	24	0	0	21	3
	100 mg/L	0	0	24	0	0	24
	Specificity (%)	100	100	100	100	100	87.5
	Sensitivity (%)	100	100	100	87.5	87.5	100
	Accuracy (%)	100			91.67		
PVC	0 mg/L	24	0	0	22	2	0
	10 mg/L	0	24	0	0	24	0
	100 mg/L	0	0	24	0	0	24
	Specificity (%)	100	100	100	100	95.83	100
	Sensitivity (%)	100	100	100	91.67	100	100
	Accuracy (%)	100			97.22		

### 3.4 Model explanation

**Figure 8:**
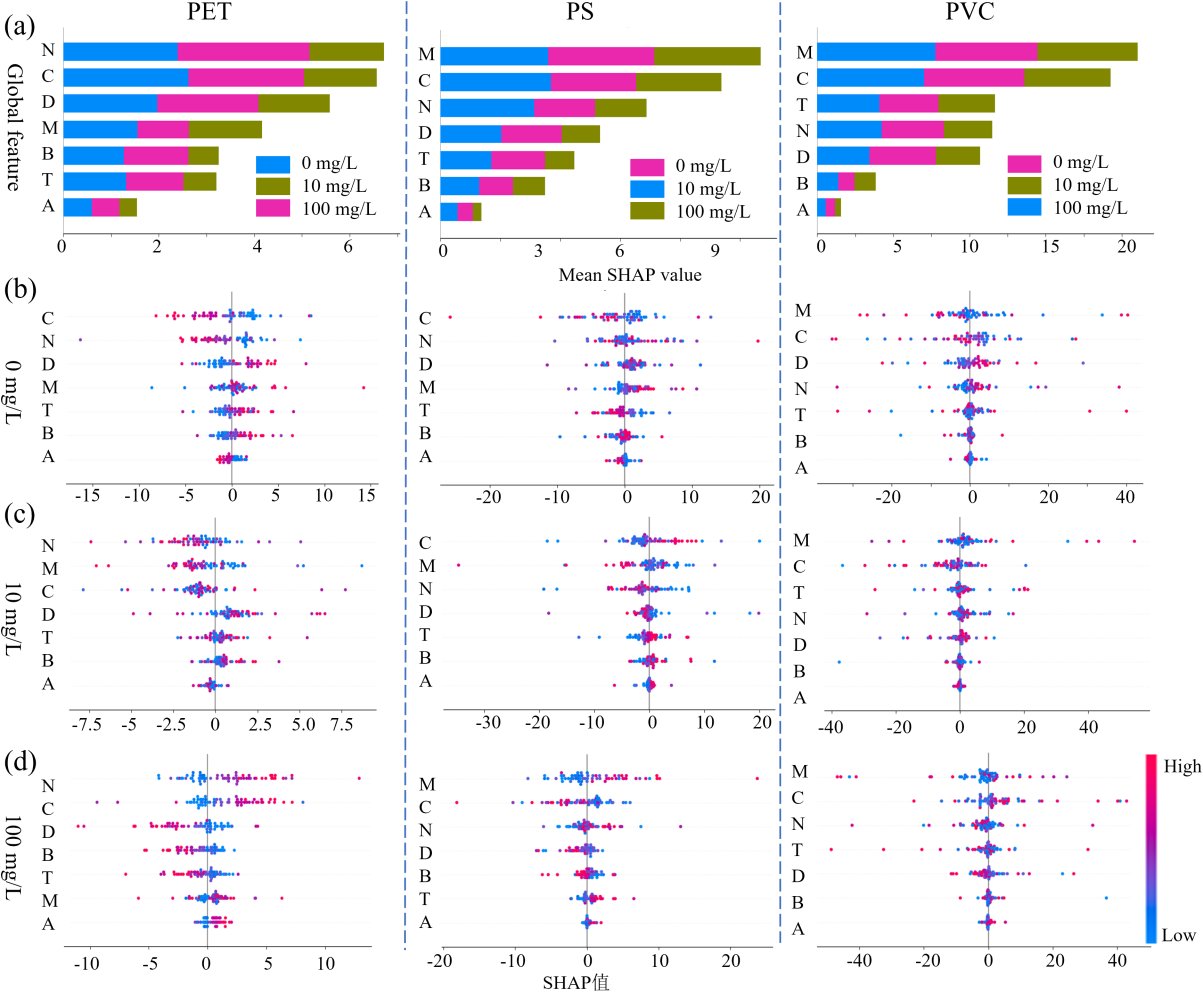
Feature importance distribution based on SHAP values for (a) the overall dataset, (b) the control group, (c) the 10 mg/L group, and (d) the 100 mg/L group.

Due to the peak method achieved better model detection results, the SHAP method was adopted to analyze the important variables in the model. The importance distribution of characteristic fluorescent markers of root exudates is shown in **Figure 8**. The first row shows the overall importance distribution of stress concentration detection, and the second to fourth rows show the importance distribution of single categories with stress concentrations of 0,10, and 100mg/L. For the PET MP detection, the SHAP value of phytoplankton related substances (N) and visible humic acid (C) was the highest, indicating that PET may stimulate root exudates to release ester bond hydrolysis products and promote alginic bilin-like substances (N) to alleviate oxidative stress. Meanwhile, the complexation of humic acid (C) may be involved in the surface modification of PET particles to reduce their toxicity. In the PS MP detection, the dominant positions of Marine humic acid (M) and visible humic acid (C) reflect that the benzene ring structure of PS may induce the root system to secrete humic acid components related to the degradation of aromatic rings, and adsorb PS particles through π-π bond interactions. In the detection of PVC MP, tryptophan (T) and Marine humic acid (M) are more relied upon, suggesting that the chloride ions released by PVC may interfere with the tryptophan metabolic pathway, while promoting the generation of halogenated humic acid (M) to facilitate the synthesis of chlorine radicals.

In the single concentration importance distribution (**Figure 8b-d**), the contribution of soil fulvic acid (D) decreased under high concentration (100 mg/L) stress, possibly because high concentration MPs inhibited the interaction between roots and soil microorganisms and reduced the biosynthesis of fulvic acid (D). The contribution of tyrosine (B) at low concentrations (10 mg/L) indicates its function as an early stress signaling molecule. Overall, it can be seen that humic acid (C) and Marine humic acid (M) both make significant contributions. The core mechanism may involve: The carboxyl and phenolic hydroxyl functional groups of humic acid interact with the surface of MPs through hydrogen bonds/coordination bonds, changing their agglomeration state; The formation of humic acid-MPs complexes affects the root system’s perception of MPs and the transmission of defense signals. Furthermore, the universal importance of tryptophan (T) suggests that it may coordinate root development and stress response by regulating the auxin signaling pathway.

## 4 Conclusions

This study proposed an interpretable and non-destructive detection method for MPs stress in rice seedlings using EEMF spectra of root exudates combined with deep learning. EEMF spectra captured significant alterations in root exudates under MPs stress, showing enhanced fluorescence intensity with increasing MPs concentrations. Characteristic fluorescent substances (humic acid-like, amino acid-like, and fulvic acid-like components) were identified through peak analysis and PARAFAC decomposition, revealing reduced relative concentrations at low MPs levels (10 mg/L) and elevated concentrations at high levels (100 mg/L) compared to controls. An optimized Vision Transformer (VIT) model using EEMF spectra, characteristic emission spectra, and fluorescence features achieved 100% classification accuracy. SHAP interpretability analysis identified humic acid-like (C) and marine humic acid-like (M) components as primary biomarkers for MPs detection.

Beyond methodological innovation, this research delivers tangible industrial and societal value. It establishes a field-deployable framework for early stress diagnosis in crops, enabling proactive interventions to safeguard food security. By eliminating destructive sampling and complex lab procedures, the approach offers agricultural and environmental monitoring industries a rapid, cost-effective solution for detecting emerging contaminants. Future work will transition this technology from controlled hydroponic conditions to complex field environments (soil systems, variable climates, and outdoor settings), accelerating its practical adoption for precision agriculture and ecosystem health assessment.

## Data Availability

The raw data supporting the conclusions of this article will be made available by the authors, without undue reservation.
